# Magnetic beads as intravesical foreign bodies in children: our clinical experience

**DOI:** 10.3389/fped.2025.1439854

**Published:** 2025-01-15

**Authors:** Yefeng Zeng, Shaogang Huang, Zhilin Yang, Xianbin Gu, Xuerui Sun, Pengyu Chen, Shoulin Li

**Affiliations:** ^1^ Department of Urology, Shenzhen Children’s Hospital, China Medical University, Shenzhen, Guangdong, China; ^2^ Department of Urology, Shenzhen Pediatrics Instutute of Shantou University Medical College, Shenzhen, China; ^3^ Department of Urology, Shenzhen Children’s Hospital, Shenzhen, Guangdong, China

**Keywords:** magnetic beads, intravesical foreign bodies, children, cystoscopy, treatment

## Abstract

**Purpose:**

To analyze the clinical data of five patients involving intravesical magnetic beads, summarizing diagnostic and therapeutic experiences.

**Methods:**

From January 2018 to November 2023, five pediatric patients were treated for intravesical magnetic beads at Shenzhen Children's Hospital. We retrospectively reviewed and analyzed the records of these patients, including demographic characteristics, clinical symptoms, imaging studies, and treatment methods.

**Results:**

All intravesical magnetic beads were retrieved from patients’ bladders. The patients ranged from 12 to 14 years, with a mean age of 13 years. None of them had a history of psychiatric disorders. Depending on the number of magnetic beads, their aggregation state, and the time since insertion, Three cases were successfully retrieved via cystoscopy, one via pneumovesicoscopy, and another via open surgery. No complications were observed during the postoperative follow-up.

**Conclusion:**

Magnetic beads are a relatively rare type of intravesical foreign bodies and should be surgically removed as soon as possible. Cystoscopy is the first method for both diagnosis and treatment. When magnetic beads cannot be retrieved via cystoscopy, pneumovesicoscopy may be a viable option for the retrieval of foreign bodies. When endoscopic techniques are unsuitable or have failed, open surgery is a necessary option.

## Introduction

Intravesical foreign bodies are considered to be relatively rare in children but typically occur in adolescents (1). These individuals may insert foreign bodies into the urethra out of sexual curiosity but fail to remove them completely, resulting in the foreign bodies remaining in the bladder (2). A wide variety of objects have been found in the bladder. However magnetic beads are relatively uncommon as intravesical foreign bodies (3). Magnetic beads, with their smooth surfaces and strong magnetic attraction to each other, can easily clump together into masses after entering the bladder through the urethra, making them difficult to remove without timely surgical intervention. Research on intravesical magnetic beads in children is limited. This study retrospectively analyzed the clinical records of five pediatric patients with intravesical foreign bodies treated in our urology department, summarizing diagnostic and therapeutic experiences.

## Materials and methods

From January 2018 to November 2023, five pediatric patients were treated for intravesical magnetic beads at Shenzhen Children's Hospital, China. We retrospectively reviewed and analyzed the records of these patients, including demographic characteristics, clinical symptoms, imaging studies, and treatment methods (Table 1).

**Table 1:** Clinical data of patients.

	Age (years)	Gender	Complaints	Duration	The number of magnetic balls	Retrieval method	Surgery time (h)	Hospitalization duration(days)
1	12	Male	No symptoms	6 h	7	Cystoscopy	0.5	1
2	13	Male	No symptoms	2 h	31	Cystoscopy	1	3
3	14	Male	Hematuria, dysuria, and fever	8 h	59	1. Cystoscopy 2. Attempted pneumovesicoscopy 3. Conversion to open surgery	2.8	11
4	13	Male	Lower abdominal pain, and dysuria	1 day	185	Cystoscopy	2.3	7
5	13	Male	Bladder foreign body sensation	1 year	14	Pneumovesicoscopy	1	15

## Results

### Case presentation

**Figure 1:**
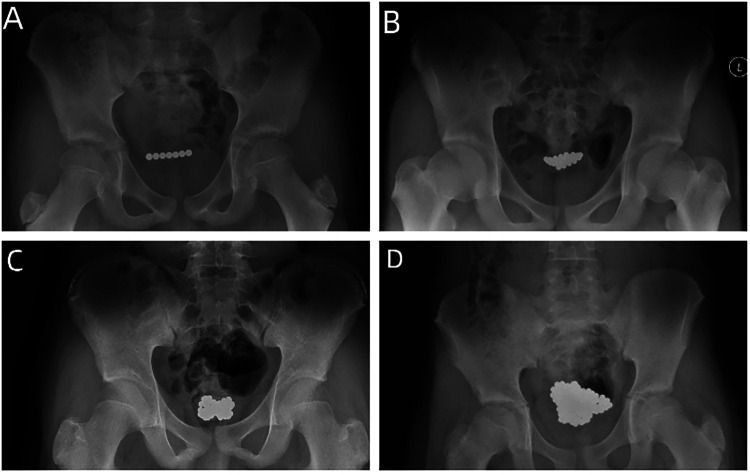
**(A)** A plain radiograph of the pelvis revealed a string of seven rounded high-density shadows in the pelvic region. **(B)** A plain radiograph of the pelvis revealed many rounded high-density shadows in a bead-like arrangement in the pelvic region. **(C)** A plain radiograph of the pelvis revealed many rounded high-density shadows in the pelvis above the pubic symphysis. **(D)** A plain radiograph of the pelvis revealed an irregular cluster of rounded high-density shadows in the pelvic region.

Case 1: A 12-year-old boy was admitted to the emergency department after he self-inserted magnetic beads into his urethra for 6 h. He presented without any symptoms. Physical examination showed no abnormalities. A plain radiograph of the pelvis revealed a string of seven rounded high-density shadows in the pelvic region (Figure 1A). Cystoscopy(13F) revealed 7 magnetic beads in the bladder. All 7 magnetic beads, each 5 mm in diameter, were successfully removed using foreign body forceps(4F) under cystoscopy. A urinary catheter was inserted post-operatively. The operation time was about 30 min. The patient was discharged on the following day. There were no complications on postoperative follow-up.

Case 2: A 13-year-old boy was admitted to the emergency department after he self-inserted magnetic beads into his urethra for 2 h. He presented without any symptoms. Physical examination showed no abnormalities. A plain radiograph of the pelvis revealed many rounded high-density shadows in a bead-like arrangement in the pelvic region (Figure 1B). Cystoscopy(13F) revealed multiple magnetic beads in the bladder. All 31 magnetic beads, each 5 mm in diameter, were successfully removed using foreign body forceps(4F) and stone baskets under cystoscopy. A urinary catheter was inserted post-operatively. The operation time was about 1 h. The patient was discharged smoothly after 3 days, with subsequent follow-up revealing no complications.

**Figure 2:**
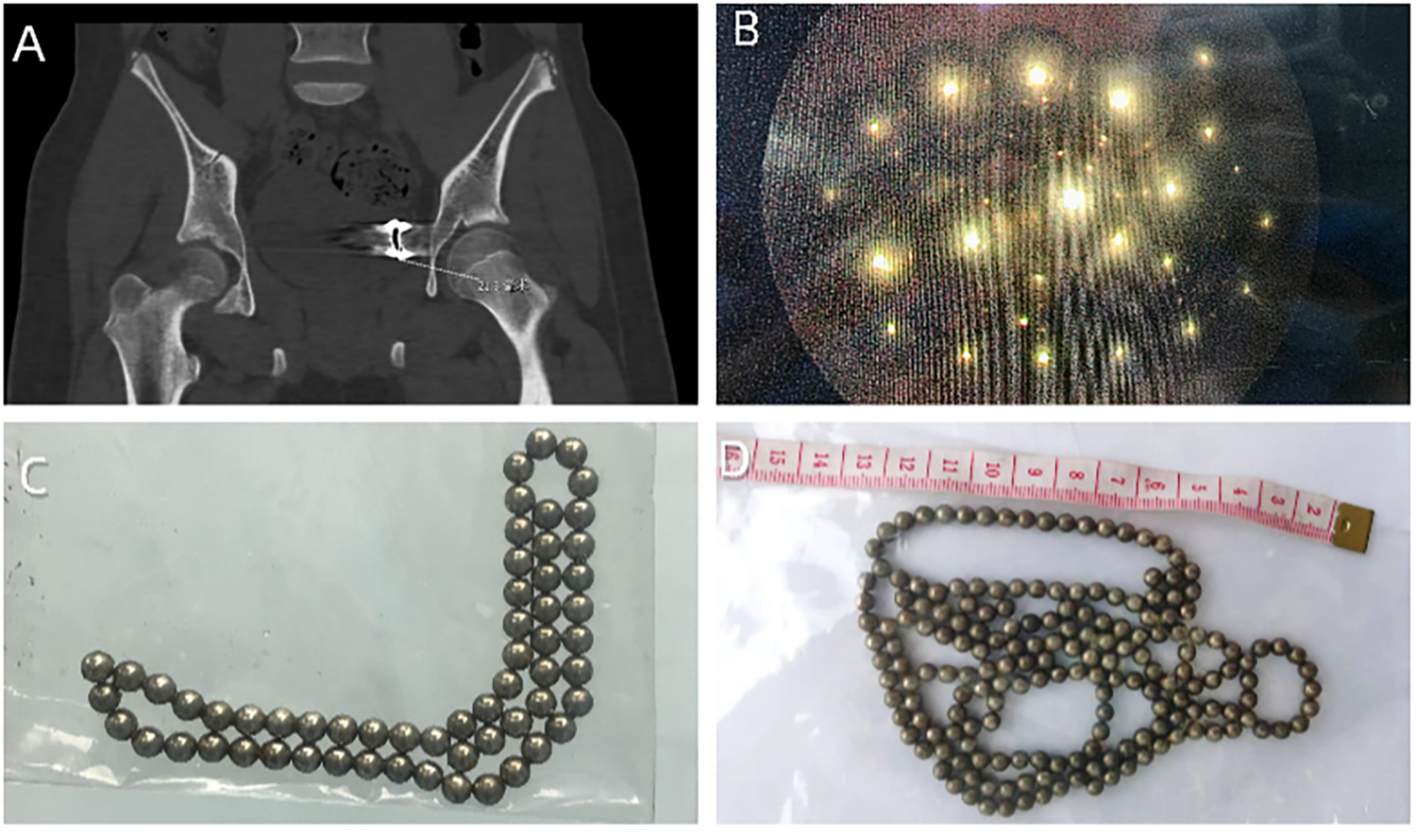
**(A)** A pelvic computed tomography (CT) scan showed a ring-shaped, bead-like high-density shadow closely adjacent to the left side of the bladder, considered to be a foreign body. **(B)** Cystoscopy revealed multiple magnetic beads in the bladder. **(C)** 59 magnetic beads of 5 mm in diameter. **(D)** 185 magnetic beads of 3 mm in diameter.

Case 3: A 14-year-old boy presented with hematuria, fever, and dysuria for 8 h after the transurethral insertion of magnetic beads. Physical examination showed mild tenderness in the bladder area. A plain radiograph of the pelvis revealed many rounded high-density shadows in the pelvis above the pubic symphysis (Figure 1C). Cystoscopy(13F) revealed multiple magnetic beads in the bladder (Figure 2B). We tried to remove the magnetic beads from the urethra via cystoscopy but failed. Then, we considered establishing pneumovesicum and removing magnetic beads via laparoscopic forceps with the help of cystoscopy. During the surgery, because gas from the trocar into the coeliac cavity and induced pneumoperitoneum, the pneumovesicum could not be maintained. Finally, We had to resort to open surgery to remove all 59 magnetic beads 5 mm in diameter (Figure 2C). A suprapubic tube was placed post-operatively. The operation time was about 2.8 h. The patient received anti-infective treatment after surgery. The patient was discharged smoothly after 11 days, with subsequent follow-up revealing no complications.

Case 4: A 13-year-old boy presented with lower abdominal pain and hematuria for 1 day after the transurethral insertion of magnetic beads. Physical examination showed no abnormalities. A plain radiograph of the pelvis revealed an irregular cluster of rounded high-density shadows in the pelvic region (Figure 1D). Cystoscopy(13F) revealed multiple magnetic beads in the bladder. All 185 magnetic beads, each 3 mm in diameter, were successfully removed using foreign body forceps(3F) and stone baskets under cystoscopy(Figure 2D). A urinary catheter was inserted post-operatively. The operation time was about 2.3 h. Prophylactic antibiotics were given for the risk of infection. The patient was discharged smoothly after 7 days, with subsequent follow-up revealing no complications.

Case 5: A 13-year-old boy was admitted due to a sensation of foreign objects in his abdomen for one week. Upon further inquiry, it was revealed that he had self-inserted magnetic beads into his urethra a year ago, which had gone untreated without any symptoms during that period. The urine culture indicated the presence of staphylococcus epidermidis. A pelvic computed tomography(CT) scan showed a ring-shaped, bead-like high-density shadow closely adjacent to the left side of the bladder, considered to be a foreign body (Figure 2A). Physical examination showed mild tenderness in the bladder area. Cystoscopy(12F) revealed multiple magnetic beads adsorbed into a mass in the bladder. At first, We tried to remove the magnetic beads via cystoscopy from the urethra but failed. Subsequently, a laparoscopy port was introduced in the bladder using cystoscopic guidance, and pneumovesicum was created. Finally, all 14 magnetic balls 5 mm in diameter were removed using laparoscopic forceps with the help of cystoscopy. The operation time was about 1 h. The patient received anti-infective treatment after surgery. A suprapubic tube was placed post-operatively. The patient was discharged smoothly after 15 days. There were no complications on postoperative follow-up.

All intravesical magnetic beads were retrieved from patients’ bladders. The patients ranged from 12 to 14 years, with a mean age of 13 years. None of them had a history of psychiatric disorders. Depending on the number of magnetic beads, their aggregation state, and the time since insertion, Three cases were successfully retrieved via cystoscopy, one via pneumovesicoscopy, and another via open surgery. No complications were observed during the postoperative follow-up.

## Discussion

Magnetic beads, finely manufactured from spherical neodymium rare-earth magnets, can be assembled into various geometric shapes using the attractive forces between their poles. They are commonly used as toys for entertainment and stress relief. Magnetic beads are most commonly accidentally ingested causing damage to the digestive system and are rarely reported as foreign bodies in the bladder (4–6). Among children, self-insertion of foreign bodies into the urethra may be due to psychiatric disorders, accidental insertions, sexual stimulation, or simple curiosity (7). Foreign bodies in the bladder are usually characterized by haematuria, frequency of urination, dysuria, and pelvic pain (8). Due to embarrassment or mental illness, patients often conceal their history of intravesical foreign bodies, leading to delayed discovery and long-term retention in the bladder. The prolonged presence of intravesical foreign bodies may result in recurrent urinary tract infections, bladder irritation signs, urinary retention, bladder perforation, peri-vesical abscesses, intestinal fistulae, vaginal fistulae, calcification or stone formation of the foreign body, pyelonephritis, hydronephrosis, and squamous cell carcinoma of the bladder (9).

The diagnosis of intravesical foreign bodies primarily relies on medical history, imaging studies, and cystoscopic examination. When abnormal imaging findings are discovered and the child shows obvious anxiety, avoidance, or refusal of external genital examination during history taking, intravesical foreign bodies should be considered to avoid misdiagnosis and treatment delays (10).

Cystoscopy is the preferred method for both diagnosing and treating intravesical foreign bodies (11). In most cases, foreign bodies can be directly removed from the bladder via cystoscopy (7, 8, 12). However, the smaller diameter of children's urethras may restrict the use of endoscopic instruments in pediatric patients (3, 11, 13, 14). The smooth surface of the magnetic beads and their mutual attraction allow them to easily clump together into a mass. This necessitates repeated insertions and removals of the cystoscope from the urethra, increasing the risk of urethral edema and bleeding. When magnetic beads cannot be removed via cystoscopy, there are different ways to remove including percutaneous nephroscope, laparoscopic extraction, pneumovesicoscopy, and open surgical removal (2, 3, 15, 16). Although open surgical removal is convenient and rapid, it is more invasive and requires an extended postoperative recovery period. Pneumovesicoscopy utilizes the body's natural pathways, results in less trauma, and potentially prevents urethral damage associated with repeated cystoscopic insertions through the urethral route. It emerges as a viable option for the retrieval of foreign bodies. The creation of a percutaneous cystostomy by an Amplatz dilator and the removal of intravesical magnetic beads via a nephroscope and a rigid grasper is also a viable option. Robey et al (3) used the way to remove 27 magnetic beads from the bladder and no complications occurred. In cases where endoscopic management is not possible, open surgery is a necessary option (2, 3, 11, 13, 15). In our study, two patients failed to have magnetic beads removed via cystoscopy. One was successfully removed via pneumovesicoscopy. The other had to resort to open surgery because gas from the trocar into the coeliac cavity induced pneumoperitoneum, causing the pneumovesicum to fail to sustain. To address this, it can insert a 3-mm Trocar or Veress needle to vent CO2 from the umbilical to reduce abdominal pressure and allow the bladder to distend appropriately (17). We need to consider many factors when addressing intravesical magnetic beads, including the diameter of the urethra of children, the number of magnetic beads, the diameter and magnet of magnetic beads, and so on. And developing a method that causes the least damage to the children. Operative complications such as urethral injury, urethral stricture, and nerve injury for impotence should be avoided (18).

Rodriguez et al (19) reported over one-third of male patients with urethral/bladder foreign bodies have significant mental health disorders. Psychiatric disorders such as schizoid personality disorder, borderline personality disorder, intoxication, and mental confusion may contribute to the self-introduction of foreign bodies into the bladder (13). Among pediatric patients, adolescents are more likely to engage in either autoerotic activity or self-harm related to underlying psychiatric illness, whereas younger children may be merely curious (3). In our study, all patients were adolescents and had no history of psychiatric disorders. They inserted the magnetic beads into the urethra because of sexual curiosity. Since children's cognitive abilities are not fully developed, it is crucial to perform psychological or psychiatric evaluations. By doing so, psychiatric problems that may have contributed to the insertion behavior can be identified and treated. Even in the absence of psychiatric illness, harm-reduction strategies may be taught to psychologically normal individuals who embrace the insertion behavior as a lifestyle preference (20).

## Conclusion

Magnetic beads are a relatively rare type of intravesical foreign bodies and should be surgically removed as soon as possible. Cystoscopy is the first method for both diagnosis and treatment. When magnetic beads cannot be retrieved via cystoscopy, pneumovesicoscopy may be a viable option for the retrieval of foreign bodies. When endoscopic techniques are unsuitable or have failed, open surgery is a necessary option.

## Data Availability

The original contributions presented in the study are included in the article/Supplementary Material, further inquiries can be directed to the corresponding authors.
